# Magnetic Phase Separation in the Oxypnictide Sr_2_Cr_1.85_Mn_1.15_As_2_O_2_

**DOI:** 10.1021/acs.inorgchem.2c00885

**Published:** 2022-08-04

**Authors:** Bor Arah, Clemens Ritter, Gavin B. G. Stenning, Abbie C. Mclaughlin

**Affiliations:** † The Chemistry Department, University of Aberdeen, Meston Walk, Aberdeen AB24 3UE, Scotland; ‡ Institut Laue-Langevin, 71 Avenue des Martyrs, 38042 Grenoble, France; § ISIS, Science and Technology Facilities Council, Rutherford Appleton Laboratory, Didcot OX11 0QX, U. K.

## Abstract

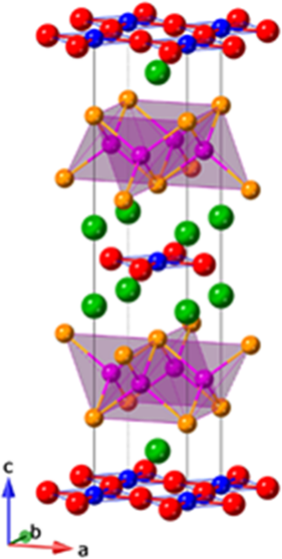

Layered Sr_2_M_3_As_2_O_2_-type oxypnictides are composed of tetrahedral M_2_Pn_2_ and square planar MO_2_ layers, the building blocks of iron-based and cuprate superconductors. To further expand our understanding of the chemical and magnetic properties of the Sr_2_Cr_3–*x*_Mn*_x_*As_2_O_2_ solid solution, Sr_2_Cr_2_MnAs_2_O_2_ has been synthesized. The compound crystallizes in the *I*4/*mmm* tetragonal space group with a refined stoichiometry of Sr_2_Cr_1.85_Mn_1.15_As_2_O_2_. The M(2) site within the M_2_Pn_2_ slab is occupied by 42.7% Cr and 57.3% Mn, and the magnetic moments order antiferromagnetically below T_N_(M2) = 540 K with a C-type antiferromagnetic structure. The M(1) site within the MO_2_ layers is fully occupied by Cr, and antiferromagnetic order is observed below T_N_(M1) = 200 K. Along *c*, there are two possible interplanar arrangements: ferromagnetic with the (1/2, 1/2, 0) propagation vector and antiferromagnetic with the (1/2, 1/2, 1/2) propagation vector. Magnetic phase separation arises so that both propagation vectors are observed below 200 K. Such magnetic phase separation has not been previously observed in Sr_2_M_3_As_2_O_2_ phases (M = Cr, Mn) and shows that there are several competing magnetic structures present in these compounds.

## Abstract 2

Layered Sr_2_M_3_As_2_O_2_-type oxypnictides are composed of tetrahedral M_2_Pn_2_ and square planar MO_2_ layers, the building blocks of iron-based and cuprate superconductors. Sr_2_Cr_1.85_Mn_1.15_As_2_O_2_ is antiferromagnetic and semiconducting. Surprisingly magnetic phase segregation is observed in the oxypnictide Sr_2_Cr_1.85_Mn_1.15_As_2_O_2_ because of competing magnetic exchange interactions along *c*.

## Introduction

Since the discovery of high-temperature superconductivity in iron oxyarsenides in 2006, layered pnictide compounds have seen increased research focus. Their superconductivity originates from the iron arsenide layers,^1^ which are present in multiple structure types such as LiFeAs,^2^ SmFeAsO_0.85,_^3^ K_0.8_Fe_2_Se_2,_^4^ CaFe_2_As_2,_^5^ α-FeSe,^6^ and (Fe_2_As_2_)[Ca_4_(Sc,Ti)_3_O_8_].^7^ It was soon discovered that upon substituting Fe for other transition metals, different phenomena emerge such as giant magnetoresistance in (La,Nd)MnAsO,^8^ a spin reorientation transition in CeMnAsO,^9^ colossal magnetoresistance in NdMnAsO_1–*x*_F_*x*,_^10^ persistent short-range order above T_N_ in LaMnAsO,^11^ p-type semiconductivity in LaCuChO (Ch = S, Se, Te),^12^ and competing spin-density-wave magnetism and superconductivity in EuFe_2_As_2_.^13^ Studying these materials could lead to advances in high-field applications and electronic data storage and open additional avenues to determine the exact mechanisms of unconventional superconductivity.

The oxyarsenides A_2_M_2_M’As_2_O_2_ (M and M’ represent different transition metals) are especially interesting, as they contain key structural elements of both the iron arsenide and cuprate superconductors. These materials crystallize in the tetragonal crystal system, space group *I*4/*mmm*, and are composed of square planar M(1)O_2_ layers and tetrahedral M(2)_2_As_2_ layers. These layers are stacked on top of each other perpendicular to the crystallographic *c* axis and are separated by layers of alkali-earth cations (A^2+^). Several oxyarsenides, specifically A_2_M_3–*x*_M’*_x_*As_2_O_2_ (A = Ba, Sr; M*_x_* = Mn_3,_^14^ Cr_3,_^15−17^ Cr_0.77_Mn_2.23,_^18^ MnZn_2,_^19,20^ Mn_2_Cu,^19^ Zn_3_^21^), have been synthesized and investigated. While Sr_2_Fe_2_CuAs_2_O_2_ should be the most interesting compound to study, being composed of the cuprate (CuO_2_) and iron-based (Fe_2_As_2_) superconductor superconducting layers, it was reported that syntheses did not yield a single-phase product.^19^ However, it was discovered that CrAs becomes superconducting under pressure as the antiferromagnetic ordering is quenched^22^ and that superconductivity could potentially be induced in Sr_2_Cr_3_As_2_O_2_, either with pressure or with chemical doping.^17^ It was also determined that MnP becomes superconducting at ∼8 GPa.^23^ This prompted our exploration of compounds within the series Sr_2_Cr_3–*x*_Mn*_x_*As_2_O_2_.

Very different magnetic structures have been reported for Sr_2_Mn_3_As_2_O_2,_^14,19^ Sr_2_Cr_3_As_2_O_2,_^16^ and Sr_2_Cr_0.77_Mn_2.23_As_2_O_2._^18^ Sr_2_Cr_3_As_2_O_2_ exhibits antiferromagnetic ordering of the M(2) sites below 590.3 K, with the propagation vector k = (1, 0, 0), and the moments aligned parallel to the *c* axis. Below 291 K, the M(1) sites order antiferromagnetically, with a K_2_NiF_4_-type antiferromagnetic structure and propagation vector k = (1/2, 1/2, 0) (moments aligned parallel to *c*), which causes a spin-flop of the M(2) spins onto the *ab* plane.^16^ The Sr_2_Mn_3_As_2_O_2_ compound exhibits G-type antiferromagnetic ordering with the propagation vector k = 0 of the M(2) sites below 340 K with spins aligned parallel to the *c* axis and short-range two-dimensional magnetic correlations below 75 K so that the M(1) sublattice does not exhibit long-range magnetic order down to 4 K. Additional peaks were observed at 4 K but could not be satisfactorily modeled.^14,19^ The mixed transition-metal compound Sr_2_Cr_0.77_Mn_2.23_As_2_O_2_ exhibits G-type antiferromagnetic ordering of the M(2) sites with k = 0 between 410 and 167 K (with moments along the *c* axis), and antiferromagnetic ordering of the M(1) sites below 167 K (moments along the *c* axis) with k = (1/2, 1/2, 0), which causes a reorientation of the M(2) moments to a C-type antiferromagnetic arrangement (moments along the *c* axis) with k = (1, 0, 0).^18^

The magnetic transition temperatures in these compounds are also affected by the transition-metal stoichiometry with T_N_ decreasing as the Cr:Mn ratio decreases.^19^ Xu et al.^15^ have recently discussed the interlayer coupling of magnetic sublattices in Sr_2_Cr_3_As_2_O_2_, prompted by their observation of a spin-flop transition of the M(2) site magnetic moments upon the emergence of long-range order of the M(1) site moments, which was also reported by Liu et al.^16^ A similar (field-induced) phenomenon has also recently been observed in EuMnBi_2_^24^ and coupling of the magnetic sublattices has been observed in the 1111-type compounds such as SmFeAsO,^25^ CeMnAsO,^26^ and PrMnSbO.^27^ However, in these examples, both magnetic sublattices have the same propagation vector. The coupling of magnetic lattices with different propagation vectors should not occur in principle; however, it seems that A_2_M_2_M’As_2_O_2_ oxypnictides exhibit this phenomenon.^15^ The spin reorientation and magnetic coupling were attributed to an antisymmetric exchange (Dzyaloshinskii–Moriya interaction), however, no significant accompanying structural distortion could be determined.^15^

Since we have observed a similar spin-flip transition in Sr_2_Cr_0.77_Mn_2.23_As_2_O_2,_^18^ we have started to explore the magnetic structures of other members of this solid solution series to better understand the interactions between the layers. We have now synthesized Sr_2_Cr_2_MnAs_2_O_2_, and here we report the crystal structure and changes in the magnetic structure with temperature.

## Experimental Section

A polycrystalline sample of the nominal stoichiometry Sr_2_Cr_2_MnAs_2_O_2_ was prepared using a standard solid-state synthesis on a 1.2 g scale. The starting materials used were Mn (99.99%, Aldrich), Cr (≥99%, Aldrich), As (99.999%, Aldrich), and SrCO_3_ (99.9 + %, Aldrich). SrO was prepared by heating SrCO_3_ at 1250 °C for 12 h, followed by quenching and immediate use. Stoichiometric amounts of Mn, Cr, and As were mixed with a nonstoichiometric amount of SrO (2% deficiency). In agreement with the reported syntheses of Sr_2_Cr_3_As_2_O_2_^16,17^ and Sr_2_Cr_0.77_Mn_2.23_As_2_O_2,_^18^ we have observed that the deficiency increased phase purity. All starting materials were mixed and ground in an inert atmosphere (N_2_, UN1066 BOC) environment using an agate mortar and pestle. The mixture was pelleted and inserted into a Ta crucible (foil 0.05 mm, ≥99.9%, Aldrich), which was sealed in a quartz tube under vacuum. The pellets were heated at 1080 °C for 72 h twice, with intermediate regrinding.

Room-temperature powder X-ray diffraction was performed on a PANalytical Empyrean Powder diffractometer. Patterns were recorded using Cu Kα1 radiation, in the range of 5° < 2θ < 100°, with a step size of 0.01313°.

Variable temperature neutron diffraction experiments were performed on the D1B and D2B diffractometers at the Institut Laue-Langevin in Grenoble, France (ILL). Approximately, 1.5 g of the sample powder was inserted into a 9 mm vanadium can. Data were recorded on the D1B diffractometer with λ = 2.512 Å, with a temperature range of 1.5–420 K upon heating at the rate of 1 K/170 s and 375–673 K on heating at the rate of 1 K/200 s, separated into 600 s snapshots (3.5–3 K range, respectively). Further patterns were recorded on the D2B diffractometer with λ = 1.594 Å at 1.5, 50, 100, 150, 200, 250, 300, 350, 400, 450, 500, and 600 K. Data were collected for 2.5 h at each temperature step.

The temperature dependence of the magnetic susceptibility was performed with a Quantum Design Superconducting Quantum Interference Device (SQUID) magnetometer. Field-cooled (FC) and zero-field-cooled (ZFC) measurements were recorded in a field of 1000 Oe between 4 and 340 K. The temperature dependence of the electrical resistance was performed with a Quantum Design physical property measurement system (PPMS). Measurements were recorded on heating between 4 and 340 K.

## Results and Discussion

**Figure 1:**
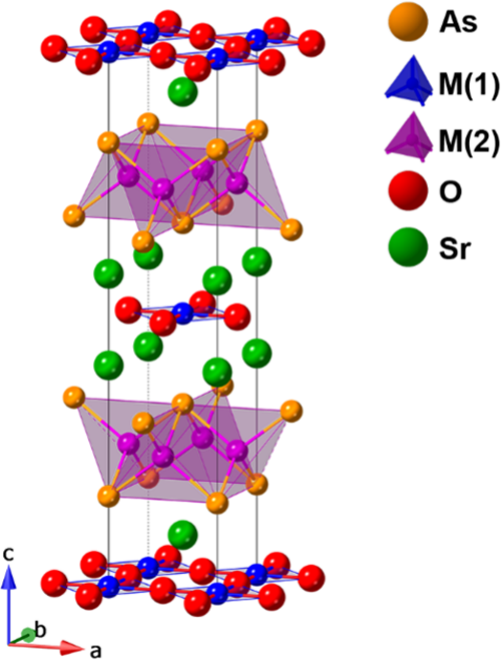
Crystal structure of Sr_2_Cr_1.85_Mn_1.15_As_2_O_2_ showing the alternating M(1) CuO_2_-type and M(2) FeAs-type layers along *c*.

Sr_2_Cr_2_MnAs_2_O_2_ crystallizes in the tetragonal crystal system with the space group *I*4/*mmm*. The unit cell of Sr_2_Cr_2_MnAs_2_O_2_ is composed of M(1)O_2_ planes and M(2)_2_As_2_ layers, separated by Sr^2+^ ions, as shown in Figure 1. Initial structural analysis was performed using Rietveld refinement on laboratory X-ray powder diffraction patterns. These were refined using a model of the Sr_2_Cr_3_As_2_O_2_ crystal cell,^15−17^ modified to Sr_2_Cr_2_MnAs_2_O_2_ by replacing half of the M(2) site Cr occupancy with Mn. The patterns could be indexed well on the tetragonal unit cell, with cell parameters *a* = 4.0428(1) Å and *c* = 18.9578(6) Å. A small impurity phase of Sr_2_Cr_2_AsO_3_ is observed and fitted (0.7% by mass). This impurity has previously been identified by Xu et al. during the synthesis of Sr_2_Cr_3_As_2_O_2_.^15^

**Figure 2:**
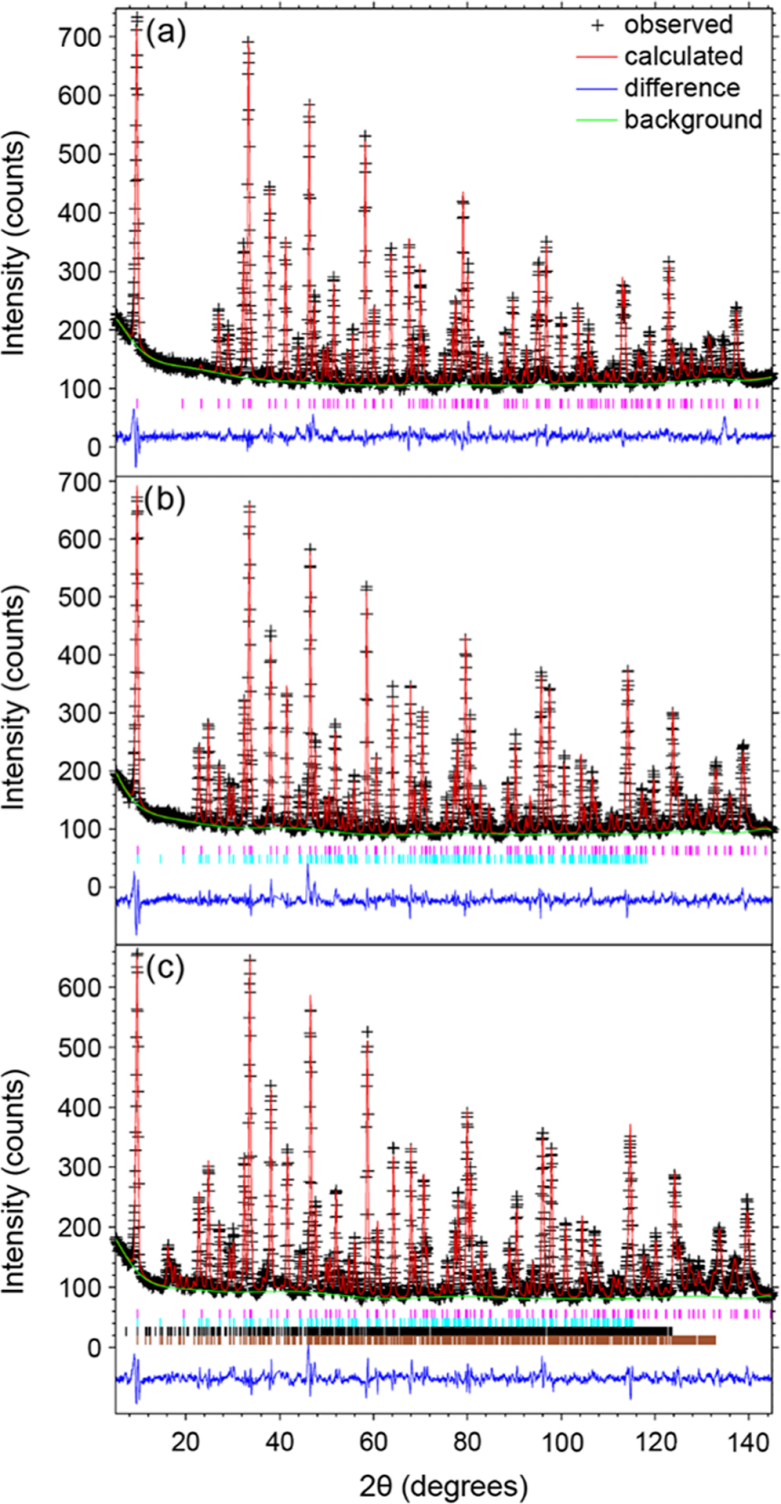
Rietveld refinements against high-resolution neutron diffraction patterns of Sr_2_Cr_1.85_Mn_1.15_As_2_O_2_ at (a) *T* = 600 K, (b) 300 K, and (c) 1.5 K. The violet, cyan, brown, and black ticks represent the possible reflections of the nuclear, k_1_ = (1, 0, 0), k_2_ = (1/2, 1/2, 0), and k_3_ = (1/2, 1/2, 1/2) magnetic phases, respectively. The patterns were recorded on the D2B diffractometer with λ = 1.594 Å. The agreement factors from Rietveld fits are shown in Table S1.

The M(1) and M(2) transition-metal sites, located at Wyckoff positions 2a and 4d, respectively, have different local environments. The M(1) sites are in square planar or, arguably, distended octahedral coordination,^15^ and the M(2) sites are in tetrahedral coordination. Powder neutron diffraction data were recorded to determine site occupancies and magnetic ordering of Sr_2_Cr_2_MnAs_2_O_2_. Rietveld refinement was first performed on the *I*4/*mmm* model with the GSAS/EXPGUI^28,29^ software using the high-resolution diffraction pattern obtained at *T* = 600 K (Figure 2a), where no magnetic peaks were observed. The background was fitted using the Chebyshev polynomial function with 15 terms and the peaks were fitted using the pseudo-Voigt profile function. An excellent fit was obtained using the *I*4*/mmm* space group with *a* = 4.06066(4) Å and *c* = 19.1220(4) Å (χ^2^ = 2.91). The atomic displacement parameters were modeled isotopically with the U_iso_s for Cr and Mn constrained to the same value. Site occupancies were refined for all atoms and fixed to 1.0 where the refined values were within ±1% of the full occupancy. It was determined that the M(1) site is fully occupied by Cr, while the M(2) site is occupied by 42.7(4)% Cr and 57.3(4)% Mn. These values were used, with no further refinement, at all temperatures. This distribution was attributed to the effects of crystal field stabilization energy and Jahn–Teller distortion, as previously reported for Sr_2_Mn_2.23_Cr_0.77_As_2_O_2._^18^ The Cr^2+^ ion favors octahedral and square planar coordination^30^ and fills completely the square planar M(1) site. In contrast, the Mn^2+^ ion displays no site selectivity and is therefore found at the M(2) site. This is also observed in A_2_MnZn_2_As_2_O_2_ (A = Sr, Ba), where the Mn^2+^ ions occupy the M(1) site and the Zn^2+^ ions occupy the M(2) site,^31^ due to the tetrahedral site preference^30^ of Zn^2+^. Additional Rietveld refinements against selected patterns are shown in Figures 2 and S1, and the refined atomic parameters, cell parameters, and agreement factors from Rietveld fits are shown in Table S1.

Rietveld refinement shows that the stoichiometry is Sr_2_Cr_1.85_Mn_1.15_As_2_O_2_ so that, like Sr_2_Cr_0.77_Mn_2.23_As_2_O_2,_^18^ the final synthesized compound contains less Cr than expected. The unit cell parameters exhibit normal thermal expansion (Figure S2). Due to the anisotropic shape of the unit cell, thermal expansion favors the *c* axis. The unit cell therefore elongates slightly with increasing temperature, and the *c*/*a* ratio changes from 4.68316(2) at 1.5 K to 4.70907(2) at 600 K (Table S1). The M(2)–As and Cr–O bond lengths are shown in Figure S3. Both bond lengths increase upon heating, while the As–M(2)–As angles do not change significantly (Table S2). There is also no evidence of magnetostriction at any temperature.

**Figure 3:**
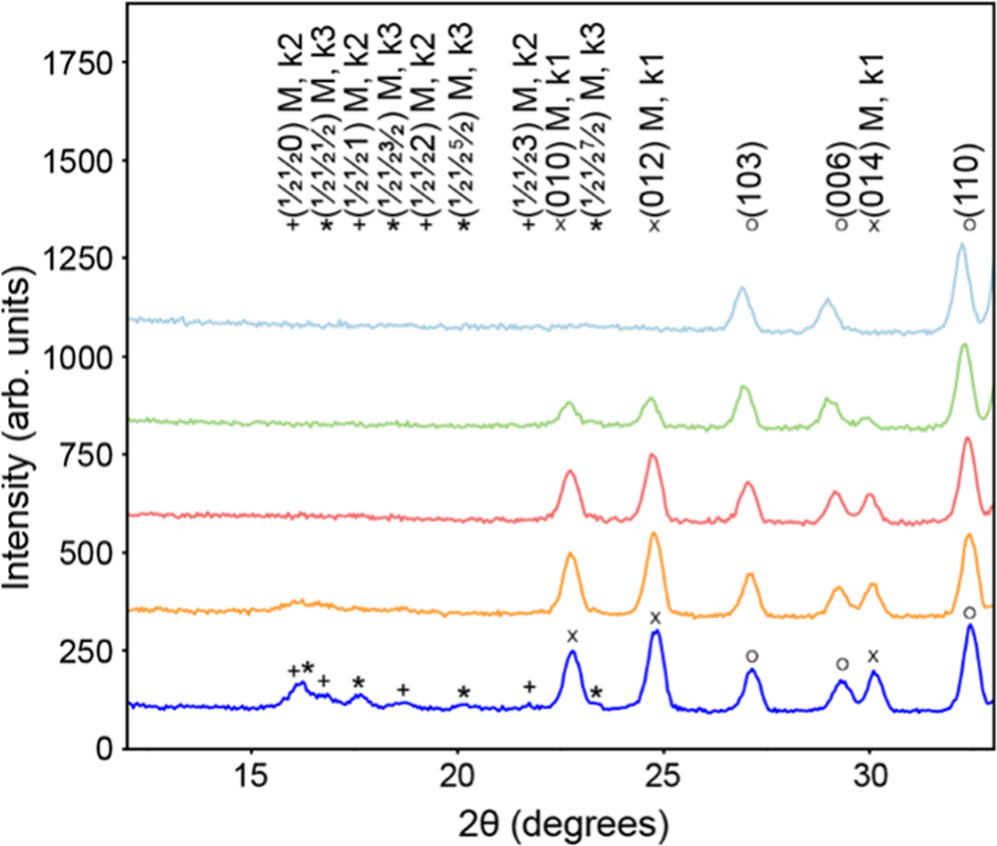
High-resolution neutron diffraction patterns at 1.5, 200, 300, 500, and 600 K (bottom to top) recorded on the D2B diffractometer with λ = 1.594 Å. The gradual appearance of magnetic diffraction peaks with a decreasing temperature is observed. The *hkl*s are marked with x, +, *, and o, for the magnetic phases with the propagation vectors k_1_ = (1, 0, 0), k_2_ = (1/2, 1/2, 0), k_3_ = (1/2, 1/2, 1/2), and the nuclear phase, respectively.

**Figure 4:**
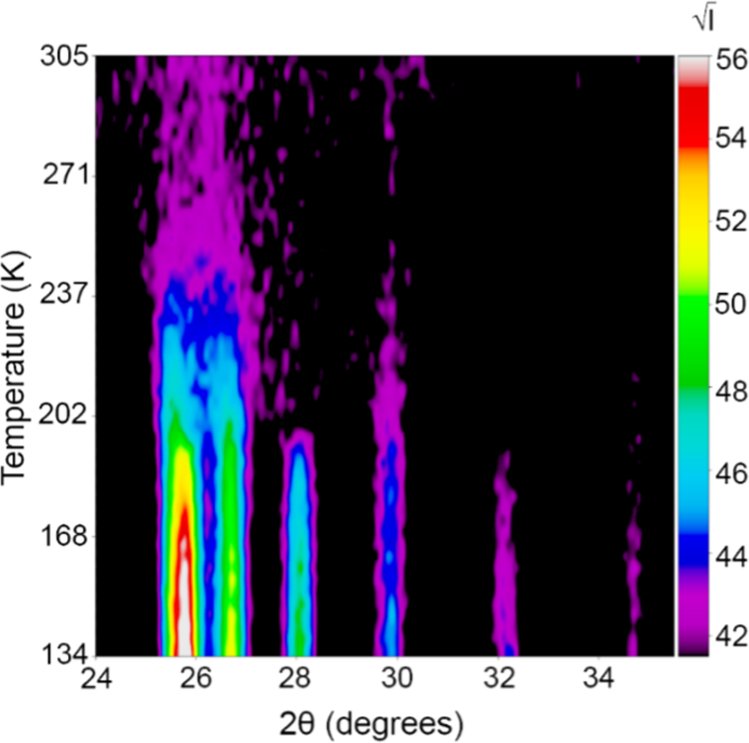
Contour plot of high-intensity data recorded on the D1B diffractometer. Data were recorded on the D1B diffractometer with λ = 2.512 Å. The intensities are presented as √I. Broad magnetic peaks are observed between 200 and 290 K, as the ordering shifts from a long range to a short range before disappearing entirely. The peaks at 28 and 32° two thetas correspond to k_3_ = (1/2, 1/2, 1/2).

**Figure 5:**
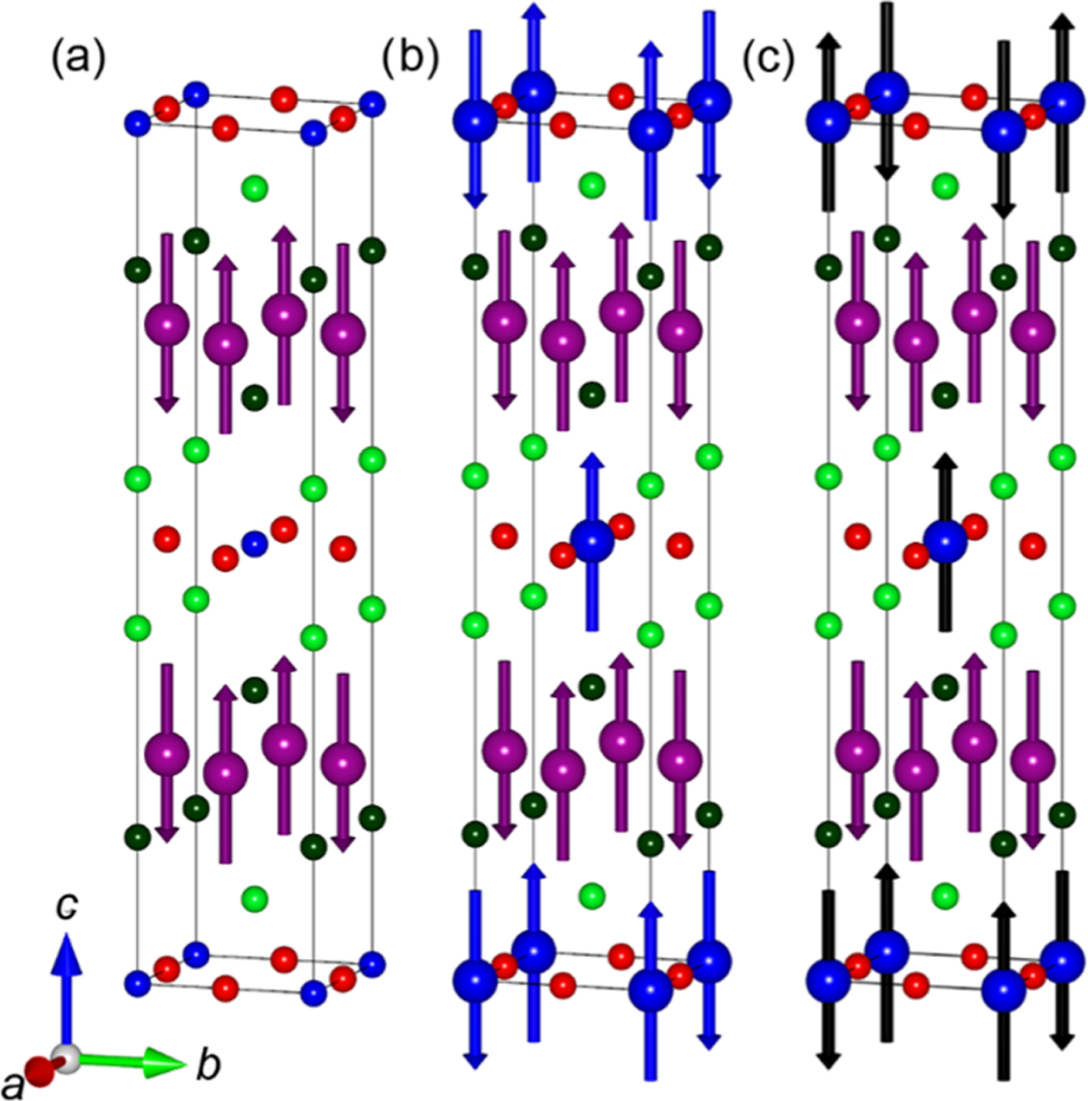
Magnetic structures of Sr_2_Cr_1.85_Mn_1.15_As_2_O_2_ below T_N_(M2) = 540 K (a) and below T_N_(M1) = 200 K (b, c). Below 200 K, all three magnetic structures are observed, with the M(1) magnetic structures depicted in (b, c) in competition. The k_1_ = (1, 0, 0), k_2_ = (1/2, 1/2, 0), and k_3_ = (1/2, 1/2, 1/2) magnetic moments are represented by violet, blue, and black arrows, respectively.

High-intensity neutron diffraction data collected on the D1B diffractometer during continuous sample heating were used to determine the onset of magnetic ordering. Additional peaks were observed below T_N_(M2) = 540 K in both the D2B data (Figure 3) and D1B data (Figure 4). These peaks could be indexed with the propagation vector k_1_ = (1, 0, 0), which corresponds to a C-type antiferromagnetic arrangement of the M(2) spins with spins aligned parallel to the *c* axis. This magnetic structure is the same as that previously reported for Sr_2_Cr_0.77_Mn_2.23_As_2_O_2_^18^ at a low temperature (Figure 5a). Below 290 K, further additional broad peaks are observed (Figures 4 and S4). Such peaks are reminiscent of short-range magnetic correlations, as observed in Sr_2_Mn_3_As_2_O_2_ below 75 K,^14^ in Ba_2_Zn_2_MnAs_2_O_2_ below 30 K,^31^ and in the pnictide layer of LaMnAsO from 360 (1) to 650 (10) K.^11^ Upon cooling below 200 K (T_N_(M1)), sharper magnetic peaks are observed, which could be indexed by two propagation vectors. The magnetic peaks can be indexed by k_2_ = (1/2, 1/2, 0), which corresponds to a K_2_NiF_4_-type antiferromagnetic structure of the M(1) site magnetic moments with moments ordered parallel to the *c* axis, as shown in Figure 5b. The magnetic peaks can also be indexed with the propagation vector k_3_ = (1/2, 1/2, 1/2) (Figures 3 and 4). The moments remain aligned parallel to the *c* axis, and the magnetic structure for this propagation vector is shown in Figure 5c. There is no change in the magnetic structure of the M(2)_2_As_2_ layer upon further cooling.

The moments on the M(1) site order with a K_2_NiF_4_-type magnetic structure. The intraplanar (*ab* plane) ordering of the magnetic moments is antiferromagnetic. Due to the body-centered tetragonal crystal structure of the compound, the coupling of the nearest neighboring MO_2_ layers’ magnetic moments is a net zero, as each atom has an equal number of ferro- and antiferromagnetically aligned neighbors. The interlayer coupling thus occurs between the next nearest neighboring (nnn) layers, which is favorable in these materials.^31^ Along *c*, there are two possible nnn interplanar arrangements: ferromagnetic with a propagation vector of (1/2, 1/2, 0) (Figure 5b) and antiferromagnetic with a propagation vector of (1/2, 1/2, 1/2) (Figure 5c).

Below 200 K, a successful fit to the experimental data was achieved with a model containing 46(4)% k_2_ = (1/2, 1/2, 0) (Figure 5b) and 54(5)% k_3_ = (1/2, 1/2, 1/2) (Figure 5c), with the k_3_ and k_2_ moments’ magnitudes constrained so that √2|*m*_k3_| = |*m*_k2_|. Hence, magnetic phase segregation is observed below 200 K. The phase fraction distribution gradually changes to 29(2)% k_2_ = (1/2, 1/2, 0) and 71(4)% k_3_ = (1/2, 1/2, 1/2) at 168 K, with no further changes down to the lowest temperature measured. During refinement, the fractions of both phases were constrained with the assumption that the entire volume of the sample exhibits ordering of the M(1) site moments. Due to the unstable nature of refining both the magnitudes and the fractions of the separate phases concurrently, they were refined separately, iterating between refining moments and scales in several steps until sufficient convergence was achieved.

**Figure 6:**
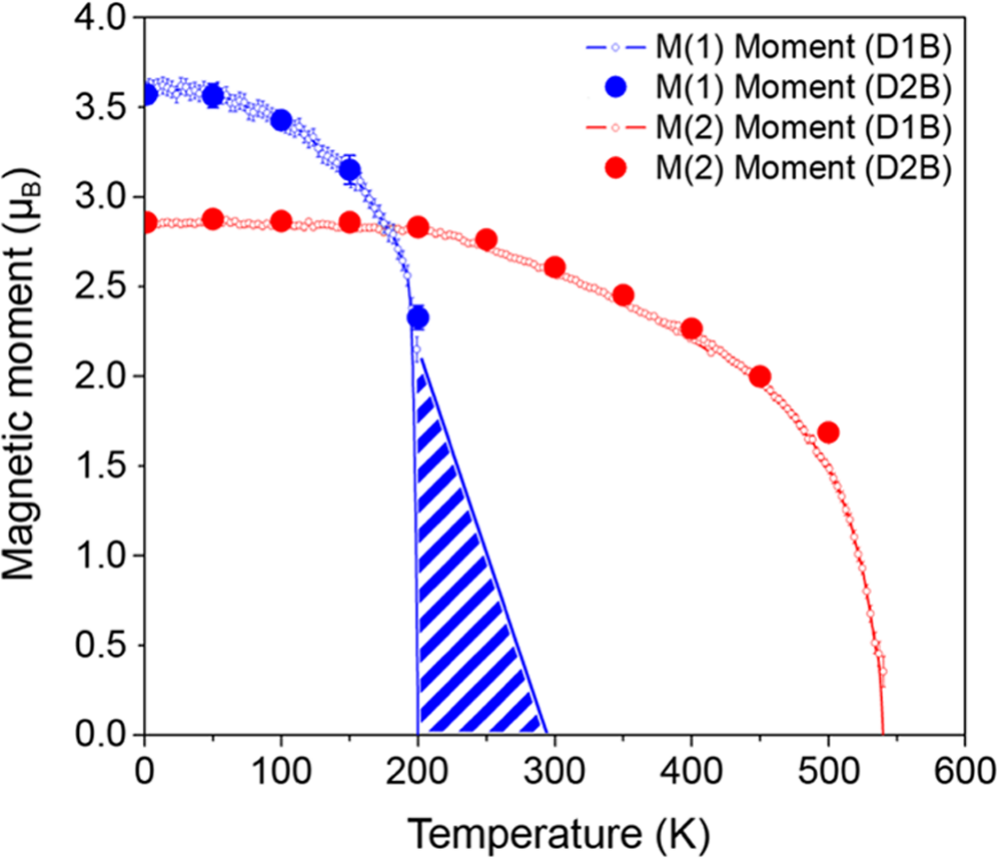
Variation of the M(1) and M(2) site magnetic moments with temperature. The smaller open symbols represent high-intensity D1B data, while the larger closed symbols represent high-resolution D2B data. The red curve represents the M(2) moments below T_N_(M2) = 540 K. Below T_N_(M1) = 200 K, the blue curve represents the M(1) moments belonging to both the magnetic phases with k_2_ = (1/2, 1/2, 0) and k_3_ = (1/2, 1/2, 1/2). The moment magnitudes were constrained as √2|*m*_k3_| = |*m*_k2_|. The blue striped area (200 K < *T* < 290 K) is an approximation of the M(1) moments where short-range magnetic order is observed.

The variation of the M(1) and M(2) site magnetic moments of Sr_2_Cr_1.85_Mn_1.15_As_2_O_2_ with temperature is shown in Figure 6. Short-range magnetic order of the M(2) moments is detected below 290 K. At 200 K, the M(1) moments order antiferromagnetically and magnetic phase separation is observed. Magnetic phase separation occurs when there is competition between magnetic ground states. Examples include the colossal magnetoresistant manganite perovskites where chemical doping on the Mn site results in competition between a charge/orbital-ordered antiferromagnetic phase and a ferromagnetic metallic phase^32^ and Ca_3_(Ru_1–*x*_Ti*_x_*)_2_O_7_ where two competing antiferromagnetic phases are observed in a narrow doping range (*x* = 0.02–0.05).^33^ Such magnetic phase separation has not been previously observed in Sr_2_M_3_As_2_O_2_ phases (M = Cr, Mn). It has been established that the intraplanar coupling constant is several orders of magnitude greater than the interlayer coupling constant, due to the long interlayer distance in these compounds. As such, interlayer coupling occurs once the correlation length within the layers reaches a sufficient length.^34^ The exchange energies for nnn layer ferromagnetic and antiferromagnetic alignment along *c* at the M(1) site would appear to be almost equivalent in this compound so that there are competing spin structures and two propagation vectors observed below T_N_(M1). Similar magnetic phase separation has previously been reported for RuSr_2_Y_1.5_Ce_0.5_Cu_2_O_10−δ_^35^ and Rb_2_MnF_4_.^36^

**Figure 7:**
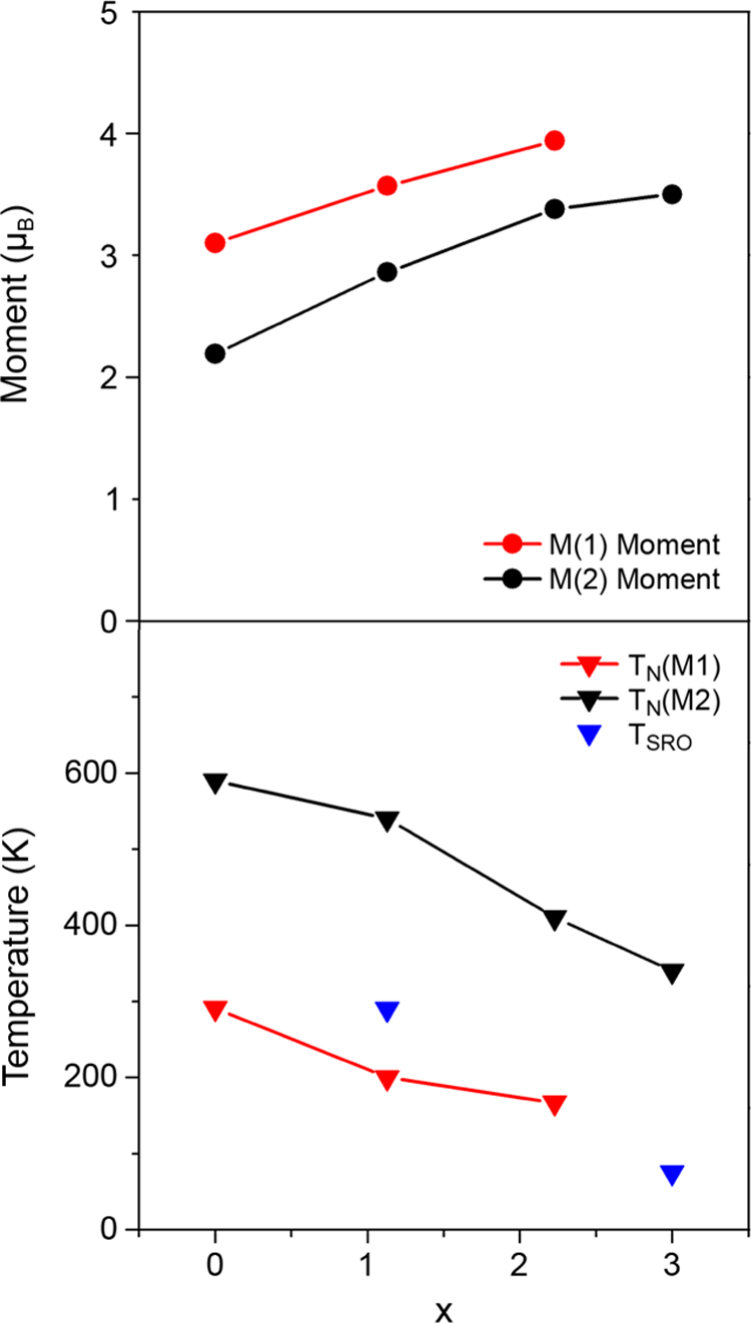
Variation of the magnetic moment magnitudes at 4 K (top) and ordering temperatures (bottom) with x in Sr_2_Cr_3–*x*_Mn*_x_*As_2_O_2_. The magnitudes of the moments at 4 K are represented by red (M(1) site) and black (M(2) site) circles. The ordering temperatures are represented by the red and black triangle marks for the long-range order of the M(1) and M(2) site magnetic moments, respectively. The blue triangle marks represent the temperatures below which the short-range order of the M(1) site magnetic moments is observed. The short-range order is observed between 290 and 200 K in Sr_2_Cr_1.85_Mn_1.15_As_2_O_2_ and below 75 K down to the lowest temperature measured in Sr_2_Mn_3_As_2_O_2_.

At 1.5 K, the saturated magnetic moments of the M(2) and M(1) sites are 2.86(2) and 3.57(5) μ_B_, respectively. It has previously been shown that there is a correlation between T_N_ at both the M(1) and M(2) sites with the Mn(1)–O and the Mn(2)–Mn(2) distances in A_2_Mn_3_Pn_2_O_2_ (A = Sr, Ba; Pn = P, As, Sb);^32^ both magnetic ordering temperatures increase as the *a* cell parameter decreases as a result of increasing superexchange (M(1)–O–M(1) and M(2)–As–M(2)) and direct exchange (M(2)–M(2)) interactions in the M(1)–O_2_ and M(2)_2_As_2_ layers. Figure S5 shows that as *x* is increased in the Sr_2_Cr_3-*x*_Mn*_x_*As_2_O_2_ solid solution, there is a linear increase in the cell volume. As a result, there is a clear reduction in the antiferromagnetic ordering temperature at both the M(1) and M(2) sites as x increases (Figure 7). This results in a lower T_N_ in Sr_2_Cr_1.85_Mn_1.15_As_2_O_2_ compared to Sr_2_Cr_3_As_2_O_2_, despite both compounds having the same M(1)O_2_ layer composition (CrO_2_). While the T_N_ in these compounds differs, the observed short-range magnetic order in Sr_2_Cr_1.85_Mn_1.15_As_2_O_2_ emerges at a similar temperature (∼290 K), as the long-range magnetic order in Sr_2_Cr_3_As_2_O_2_ (291 K). As the cell volume then decreases to 309.194(7) Å^3^ at 200 K, the correlation length in the CrO_2_ layers in Sr_2_Cr_1.85_Mn_1.15_As_2_O_2_ reaches a sufficient length for the long-range order to be established.

Due to the partially covalent nature of the M–As and M–O bonds, the magnitudes of the saturated magnetic moments are lower than the theoretical values for Cr^2+^ and Mn^2+^ (4 and 5 μ_B_, respectively) in all members of the series. However, the magnitudes of the M(2) and M(1) site moments both rise as the Mn^2+^ occupancy increases. The magnitude of the M(1)- and M(2)-ordered moments increases almost linearly with *x* in Sr_2_Cr_3–*x*_Mn*_x_*As_2_O_2_, for 0 ≤ *x* ≤ 2.23 so that there does not seem to be any effect of Mn^2+^/Cr^2+^ disorder on the overall ordered moment. The results suggest that the M(2) site moment magnitude is directly dependent on the composition of the layer, as the magnitude increases in proportion to the concentration of Mn^2+^ in the M(2)_2_As_2_ layer. The M(1) moment also increases linearly with *x*, which is surprising given that there is no change in the M(1)O_2_ layer composition from *x* = 0–1.15. This increase in the M(1) moment could be a result of the reduction in covalency as the M(1)–O bond length increases across the series.

The magnetic structures reported so far for the Sr_2_Cr_3–*x*_Mn*_x_*As_2_O_2_ series are all different.^14,15,18^ Sr_2_Cr_3_As_2_O_2_ and Sr_2_Cr_1.85_Mn_1.15_As_2_O_2_ both exhibit C-type antiferromagnetic order in the M(2)_2_As_2_ layer below T_N_(M2), whereas G-type antiferromagnetic order is observed for Sr_2_Mn_2.23_Cr_0.77_As_2_O_2_ and Sr_2_Mn_3_As_2_O_2_. This would suggest that the presence of Cr in the M(2)_2_As_2_ layer results in ferromagnetic interactions along *c*. As the order is established on the M(1) site below T_N_(M1), different magnetic structures are observed. In Sr_2_Cr_3_As_2_O_2_, the ordering of the M(1) site moments causes a spin-flop of the M(2) site moments.^15^ In Sr_2_Cr_0.77_Mn_2.23_As_2_O_2_, the ordering of the M(1) moments is followed by a spin-flip of the M(2) moments, shifting the antiferromagnetic ordering from G-type to C-type. In Sr_2_Cr_1.85_Mn_1.15_As_2_O_2_, there is no evidence of a spin-flip or -flop so there does not appear to be any coupling of the M(1) and M(2) spins. This could be driven by the sizeable cation disorder in the M(2)_2_As_2_ layer, but further research is warranted. In contrast to other members of the series, magnetic phase segregation is observed in Sr_2_Cr_1.85_Mn_1.15_As_2_O_2_ below 200 K and the two phases remain in competition down to the lowest measured temperature.

**Figure 8:**
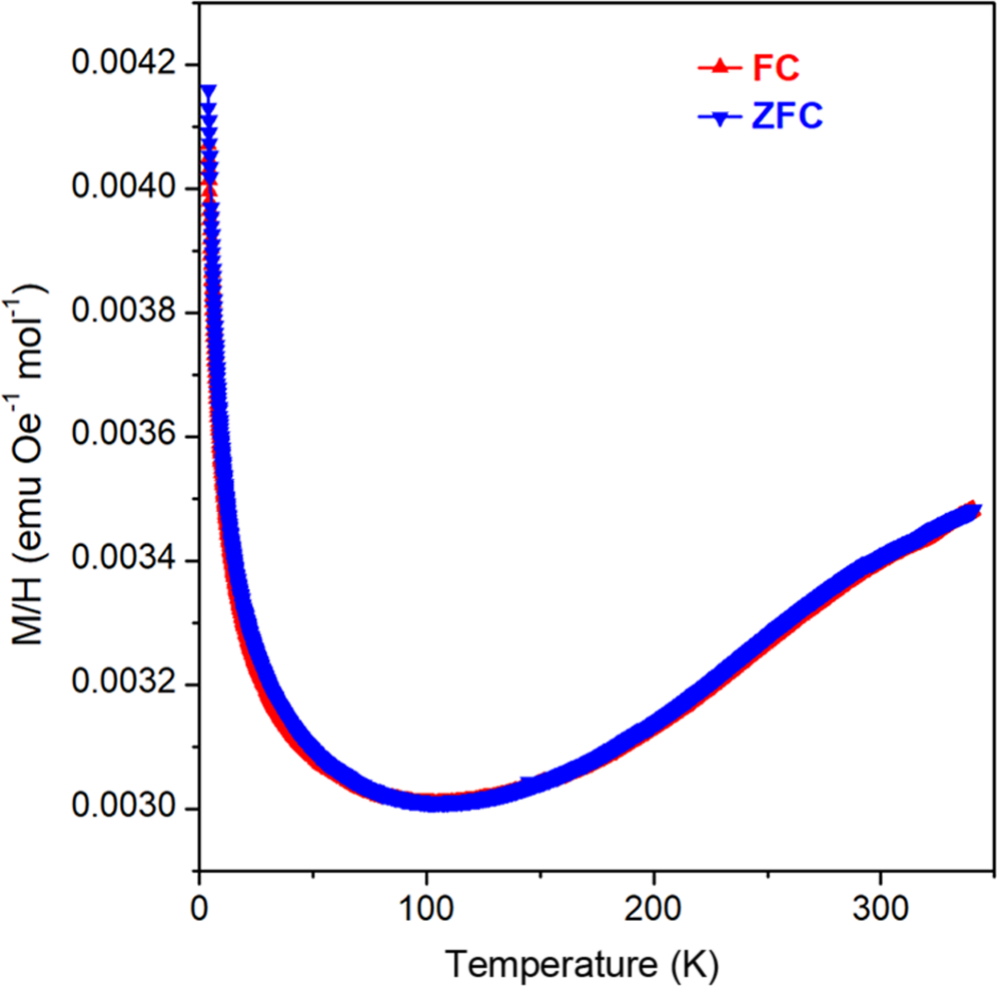
Temperature variation of the field-cooled and zero-field-cooled (FC and ZFC) susceptibility of Sr_2_Cr_1.85_Mn_1.15_As_2_O_2_ (H = 1000 Oe).

The temperature variation of the magnetic susceptibility of Sr_2_Cr_1.85_Mn_1.15_As_2_O_2_ is presented in Figure 8. The reduction in susceptibility upon cooling is a signature of the antiferromagnetic transition in the M(2)_2_As_2_ layer at T_N_(M2) = 540 K. There is no evidence of any further magnetic transitions in the susceptibility. The low-temperature magnetic transition in Sr_2_Cr_0.77_Mn_2.23_As_2_O_2_ was also not evident in the magnetic susceptibility data.^18^ There is no divergence between the FC and ZFC susceptibilities, and the upturn in the susceptibility below *T* ≈ 100 K is attributed to paramagnetic impurity spins.

**Figure 9:**
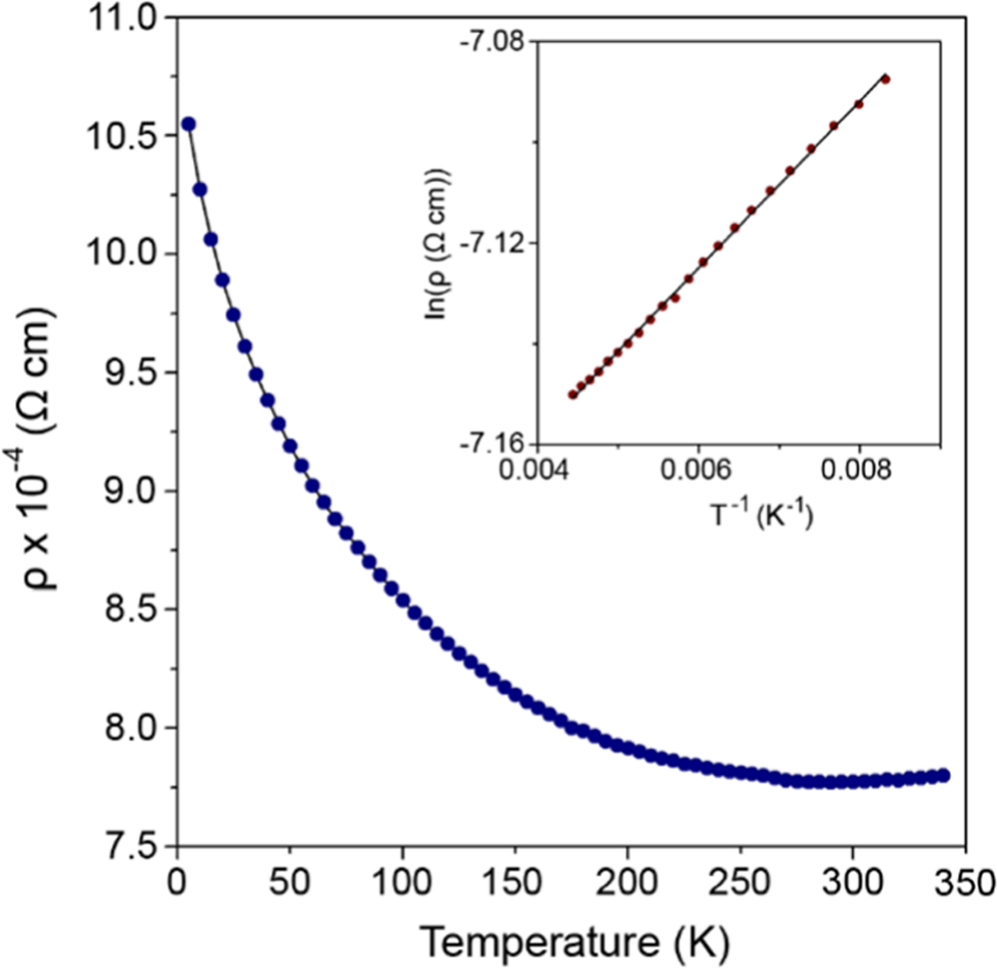
Temperature dependence of the electrical resistivity for Sr_2_Cr_1.85_Mn_1.15_As_2_O_2_. The inset shows the fit to the Arrhenius equation between 120 and 225 K.

The thermal variation of the electrical resistivity of Sr_2_Cr_1.85_Mn_1.15_As_2_O_2_ is presented in Figure 9 and exhibits semiconducting behavior. The 290 K resistivity is 7.769(5) × 10^–4^ Ω cm, which is suggestive of a highly doped semiconductor verging on metallic behavior. The resistivity can be fit to the Arrhenius equation between 120 and 225 K giving a band gap of 2.85 meV. The electronic properties of the A_2_M_2_M’As_2_O_2_ compounds are dictated by the M(2)_2_As_2_ layers.^17,18^ Cr_2_As_2_ layers exhibit metallic conductivity,^17^ while Mn_2_As_2_ layers exhibit semiconducting behavior.^18^ In the case of Sr_2_Cr_1.85_Mn_1.15_As_2_O_2_, the M(2)_2_As_2_ layers are composed of Mn_1.15_Cr_0.85_As_2_ and its resistivity exhibits a highly doped semiconductor-like temperature dependence. The conductivity of the Sr_2_Cr_3–*x*_Mn*_x_*As_2_O_2_ phases increases as the percentage of Cr on the M(2) site rises^17,18^ so that the electronic properties can be tuned by varying the ratio of Cr:Mn on the M(2) site.

## Conclusions

We have synthesized and analyzed the crystal and magnetic structures of Sr_2_Cr_1.85_Mn_1.15_As_2_O_2_, which is the fourth member of the Sr_2_Cr_3–*x*_Mn*_x_*As_2_O_2_ solid solution. The magnetic properties are distinct from the other members of the series. In Sr_2_Cr_1.85_Mn_1.15_As_2_O_2_, below T_N_(M1), magnetic phase separation is observed so that the M(1) moments order with the propagation vectors k_2_ = (1/2, 1/2, 0) and k_3_ = (1/2, 1/2, 1/2). The two phases remain in competition down to the lowest measured temperature. The competing spin structures arise as the exchange energies for nnn layer ferromagnetic and antiferromagnetic alignment at the M(1) site along *c* are almost equivalent in this compound. The results show that it is possible to tune the magnetic transition temperatures and magnetic structures by doping on the Cr site in Sr_2_Cr_3–*x*_Mn*_x_*As_2_O_2_. Substitution of Sr^2+^ for Ca^2+^ and Ba^2+^ or a variable temperature neutron diffraction at high pressure could provide further insight into the effects of interlayer distances and cell size on magnetic ordering in the Sr_2_Cr_3–*x*_Mn*_x_*As_2_O_2_ compounds.

## Supporting Information Available

It includes figures showing Rietveld refinement fits, temperature variation of cell parameters and selected bond lengths, and tables of crystallographic data (PDF)
