# Supplementary material for: Magnetic Phase Separation in the Oxypnictide Sr2Cr1.85Mn1.15As2O2
Source: Inorg Chem. 2022 Aug 4;61(32):12518–25. doi: 10.1021/acs.inorgchem.2c00885 (PMC9387525; doi:10.1021/acs.inorgchem.2c00885)
Supplement: Supplementary file 1 — ic2c00885_si_001.pdf [file ic2c00885_si_001.pdf]

Supplementary information for

Magnetic Phase Separation in the Oxypnictide $Sr_2Cr_{1.85}Mn_{1.15}As_2O_2$

Bor Arah[1], Clemens Ritter[2], Gavin B. G. Stenning[3] and Abbie C. Mclaughlin*[1]

1 The Chemistry Department, University of Aberdeen, Meston Walk, Aberdeen, AB24 3UE, Scotland.

2 Institut Laue-Langevin, 71 Avenue des Martyrs, 38042 Grenoble, France.

3 ISIS, Science and Technology Facilities Council, Rutherford Appleton Laboratory, Didcot OX11 0QX, U.K

* a.c.mclaughlin@abdn.ac.uk

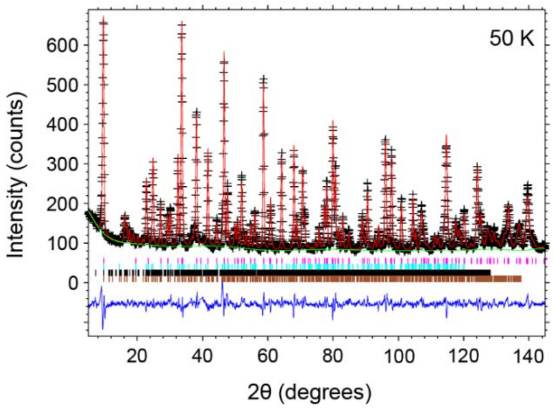

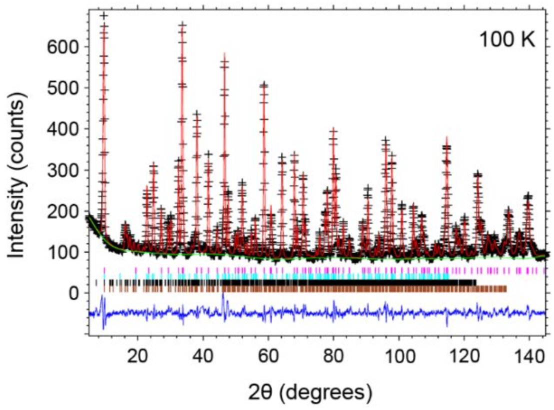

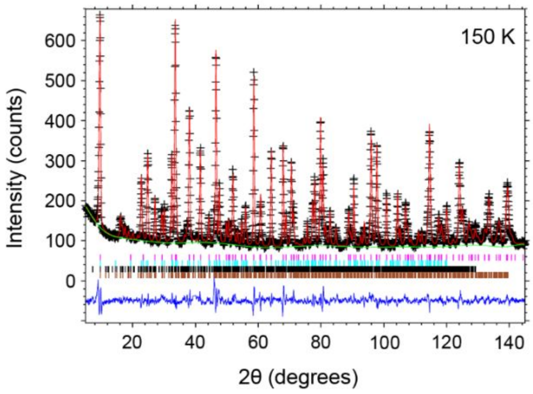

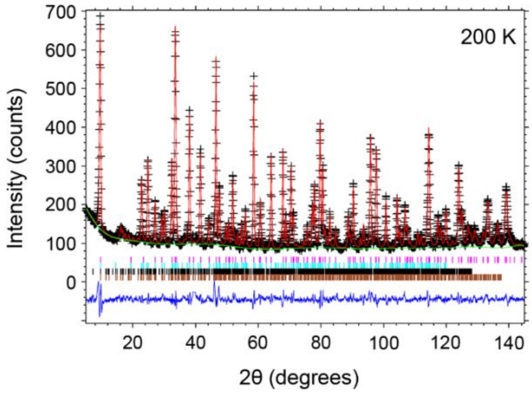

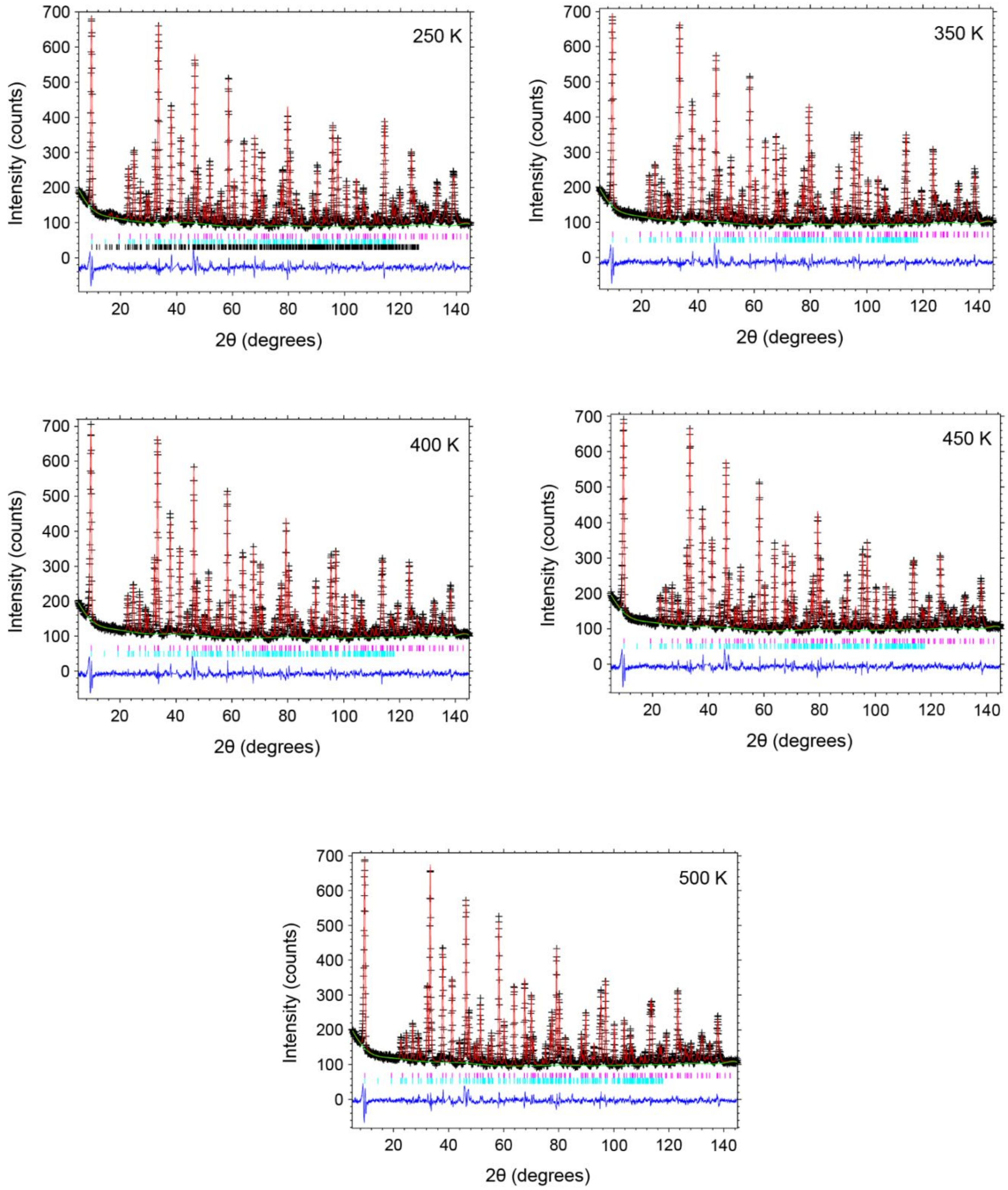

Figure S1: Additional Rietveld refinements against D2B high resolution neutron diffraction patterns of $Sr_2Cr_{1.85}Mn_{1.15}As_2O_2$ at T = 50 K, 100 K, 150 K, 200 K, 250 K, 350 K, 400 K, 450 K, and 500 K. The violet, cyan, brown, and black ticks represent the possible reflections of the nuclear, k_1 = (1, 0, 0) magnetic, k_2 = (½, ½, 0), and k_3 = (½, ½, ½) magnetic structures, respectively, in the temperature ranges where the respective magnetic moments order. The black + marks represent observed intensities, the green curve represents the background, the red curve represents the model, and the blue curve represents the difference.

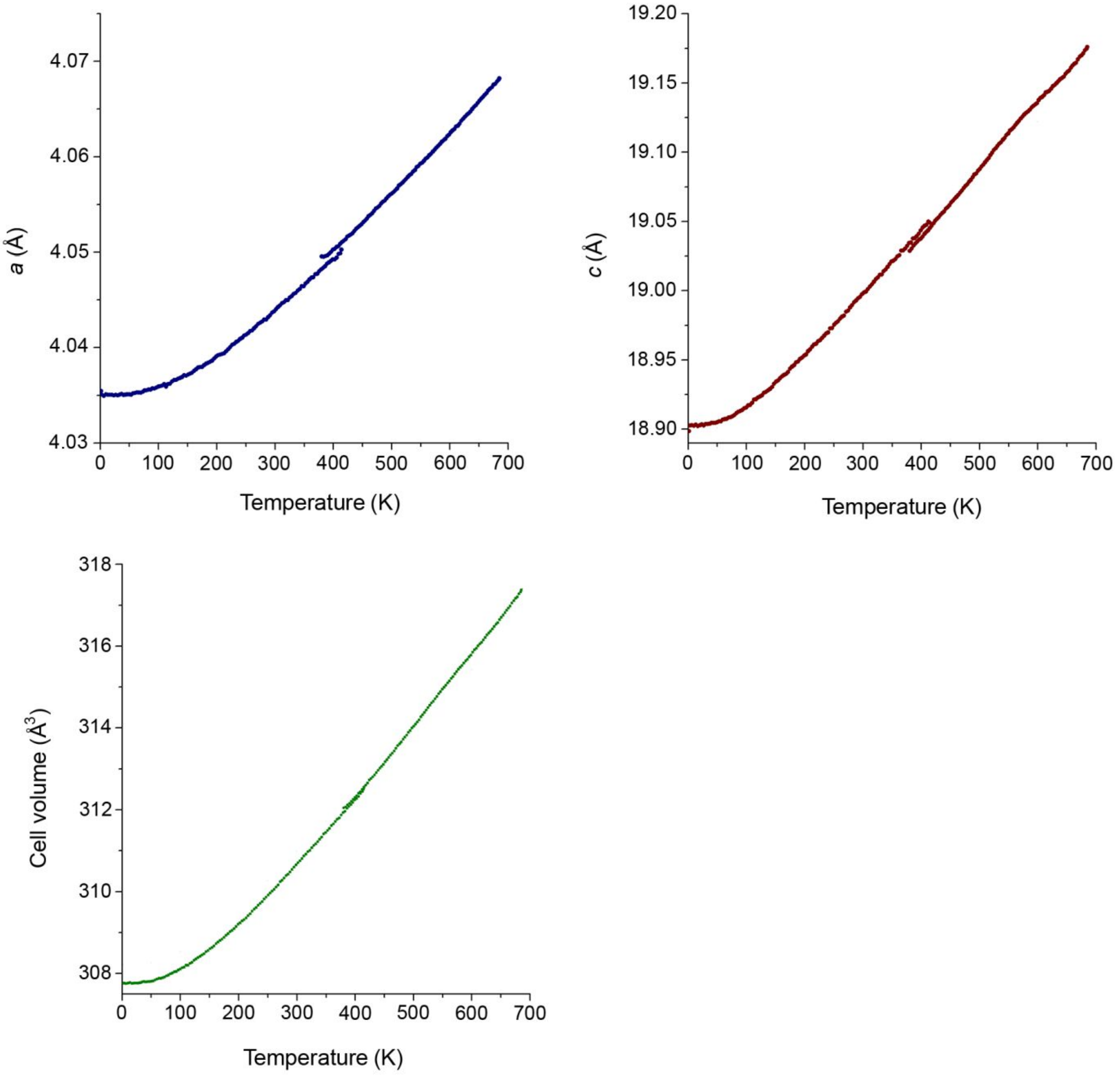

Figure S2: Cell volume, cell parameter *a*, and cell parameter *c* variation with temperature as refined against D1B high intensity neutron diffraction patterns. The data were recorded on heating. The discrepancy between the high and low temperature data sets for *a* and *c* cell parameter data can be attributed to different sample environments (a furnace and a cryofurnace, respectively).

Table S1: Refined atomic parameters, ordered moments, cell parameters, magnetic phase fractions and agreement factors from Rietveld fits against D2B high resolution diffraction data, recorded at various temperatures. M(1) is at 2*a* (0, 0, 0), M(2) is at 4*d* (0, ½, ¼), Sr and As are at 4*e* (0, 0, z), and O is at 4*c* (0, ½, 0). The data recorded at T = 600 K was used to determine site occupancies. M(1) was determined to be fully occupied by Cr, while the refined fractional occupancies for M(2) were F_{Cr} = 0.427(4) and F_{Mn} = 0.573(4).

	Temperature (K)					
	**1.5**	**50**	**100**	**150**	**200**	**250**
**Sr z**	0.41313(9)	0.41319(9)	0.41320(9)	0.41330(9)	0.41327(8)	0.41331(9)
**Sr U_{iso} (Å^2)**	0.13(6)	0.7(4)	0.17(4)	0.27(4)	0.32(4)	0.36(4)
**As z**	0.16950(9)	0.16959(9)	0.16962(9)	0.16966(9)	0.16966(8)	0.16968(9)
**As U_{iso} (Å^2)**	0.35(6)	0.34(4)	0.44(4)	0.50(4)	0.63(4)	0.79(4)
**M U_{iso} (Å^2)**	0.16(10)	0.10(9)	0.21(1)	0.20(10)	0.54(9)	0.6(1)
**O U_{iso} (Å^2)**	0.29(5)	0.19(4)	0.25(4)	0.29(4)	0.43(4)	0.48(4)
**Ordered moment M(2)**	2.86(2)	2.90(2)	2.88(2)	2.86(2)	2.87(2)	2.76(2)
**Ordered moment M(1)**	3.57(5)	3.56(7)	3.42(5)	3.15(8)	2.22(6)	1.04(8)
**k_2 = (½, ½, 0) fraction**	0.29(2)	0.29(2)	0.29(2)	0.29(2)	0.46(3)	/
**k_3 = (½, ½, ½) fraction**	0.71(4)	0.71(4)	0.71(4)	0.71(4)	0.54(5)	1
**a (Å)**	4.035437(4)	4.035448(1)	4.036189(1)	4.038032(2)	4.039119(2)	4.041326(3)
**c (Å)**	18.8986(4)	18.9011(1)	18.9119(1)	18.9239(2)	18.9521(2)	18.9745(3)
**Unit Cell Volume (Å^3)**	307.759(8)	307.802(2)	308.090(3)	308.567(3)	309.194(4)	309.898(6)
**χ^2 (%)**	3.946	3.993	3.738	3.99	3.466	3.593
**R_{wp} (%)**	5.37	5.40	5.20	5.33	4.95	5.00
**R_p (%)**	4.13	4.14	3.95	4.08	3.75	3.77
**R_{bragg} (%)**	8.12	7.41	7.09	6.91	6.63	6.42
**R_{mag} M(1) (%)**	11.15	9.54	9.72	9.79	12.70	14.18
**R_{mag} M(2) (%)**	8.16	8.27	8.96	7.32	9.25	11.36
	**Temperature (K)**					
	**300**	**350**	**400**	**450**	**500**	**600**
**Sr z**	0.41339(9)	0.4138(9)	0.41326(8)	0.41330(9)	0.41336(9)	0.41320(9)
**Sr U_{iso} (Å^2)**	0.51(4)	0.58(4)	0.61(4)	0.72(4)	0.89(4)	1.06(4)
**As z**	0.16984(9)	0.16975(9)	0.16976(9)	0.16981(9)	0.16993(9)	0.17009(9)
**As U_{iso} (Å^2)**	0.87(4)	1.02(4)	1.14(4)	1.29(4)	1.36(5)	1.69(5)
**M U_{iso} (Å^2)**	0.74(1)	0.84(9)	0.96(9)	1.04(9)	1.16(9)	1.29(9)
**O U_{iso} (Å^2)**	0.61(4)	0.70(4)	0.74(4)	0.80(4)	0.96(4)	1.15(4)
**Ordered moment M(2)**	2.61(2)	2.45(2)	2.27(2)	1.99(2)	1.68(3)	/
**Ordered moment M(1)**	/	/	/	/	/	/
**k_2 = (½, ½, 0) fraction**	/	/	/	/	/	/
**k_3 = (½, ½, ½) fraction**	/	/	/	/	/	/
***a* (Å)**	4.04379(5)	4.04641(4)	4.04905(4)	4.05167(4)	4.05430(4)	4.06067(4)
***c* (Å)**	18.9977(4)	19.0202(4)	19.0425(4)	19.0629(4)	19.0839(4)	19.1220(4)
**Unit Cell Volume (Å^3)**	310.654(7)	311.426(7)	312.199(7)	312.937(8)	313.688(7)	315.303(7)

**χ^2(%)**	3.46	3.344	3.196	3.185	3.158	2.909
**R_{wp}**	4.89	4.78	4.65	4.63	4.6	4.32
**R_p**	3.88	3.59	3.49	3.44	3.47	3.29
**R_{bragg}**	4.83	5.33	4.98	4.99	5.26	6.7
**R_{mag} M(1)**	/	/	/	/	/	/
**R_{mag} M(2)**	9.42	12.36	14.07	13.36	18.91	/

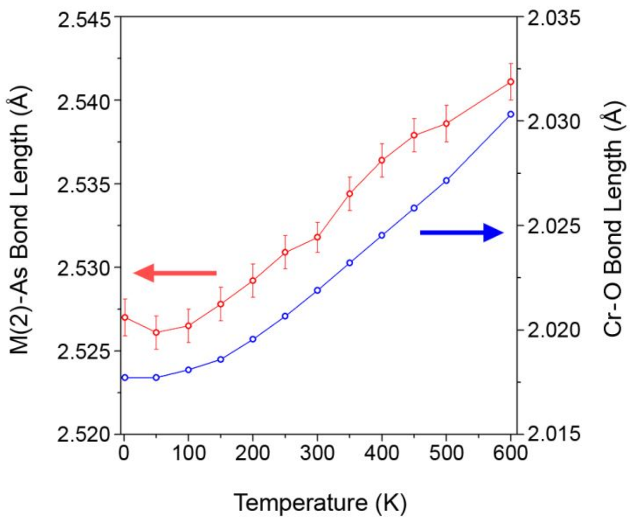

Figure S3: Variation of the M(2)-As and Cr-O bond lengths with temperature.

Table S2: Temperature variation of selected bond lengths, angles and h_{As} (the calculated distance between As and M(2) planes within the $M(2)_2As_2$ layers) from high resolution D2B data Rietveld refinement.

Temperature [K]		1.5	50	100	150	200	250
Distance [Å]	Sr-As	3.2529(14)	3.2544(13)	3.2557(13)	3.2577(13)	3.2600(13)	3.2628(13)
	Sr-O	2.6012(11)	2.6006(10)	2.6013(10)	2.6024(10)	2.6039(10)	2.6055(10)
	Sr-Cr1	3.2920(8)	3.2915(8)	3.2923(8)	3.2935(8)	3.2953(8)	3.2972(8)
	Sr-M(2)	3.6846(14)	3.6860(14)	3.6878(13)	3.6917(13)	3.6950(13)	3.6993(14)
	Cr1-O	2.01772(0)	2.01772(0)	2.01809(0)	2.01859(0)	2.01956(0)	2.02066(0)
	Cr1-As	3.2033(18)	3.2055(16)	3.2079(16)	3.2120(16)	3.2154(16)	3.2196(17)
	M(2)-As	2.5270(11)	2.5261(10)	2.5265(10)	2.5278(10)	2.5292(10)	2.5309(10)
	M(2)-M(2)	2.85348(0)	2.85349(0)	2.85402(0)	2.85471(0)	2.85609(0)	2.85765(0)
	h_{As}	1.521(1)	1.520(1)	1.520(1)	1.521(1)	1.523(1)	1.524(1)
Angle [°]	As-M(2)-As 1	105.97(6)	106.02(6)	106.03(6)	105.99(6)	105.97(6)	105.95(6)
	As-M(2)-As 2	111.250(33)	111.222(31)	111.221(30)	111.241(30)	111.249(30)	111.259(31)
Temperature [K]		300	350	400	450	500	600
Distance [Å]	Sr-As	3.2675(13)	3.2681(13)	3.2706(13)	3.2738(14)	3.2780(14)	3.2835(14)
	Sr-O	2.6068(10)	2.6104(10)	2.6128(10)	2.6145(11)	2.6159(11)	2.6224(11)
	Sr-Cr1	3.2990(8)	3.3026(8)	3.3054(8)	3.3075(8)	3.3094(8)	3.3165(9)
	Sr-M(2)	3.7044(14)	3.7065(14)	3.7100(14)	3.7141(14)	3.7187(14)	3.7231(14)
	Cr1-O	2.02189(2)	2.02321(2)	2.02453(2)	2.02583(2)	2.02715(2)	2.03033(2)
	Cr1-As	3.2266(17)	3.2287(17)	3.2326(17)	3.2371(17)	3.2429(18)	3.2524(18)
	M(2)-As	2.5312(10)	2.5344(10)	2.5364(10)	2.5379(10)	2.5386(11)	2.5411(11)
	M(2)-M(2)	2.85939(3)	2.86124(3)	2.86311(3)	2.86496(3)	2.86682(3)	2.87132(3)
	h_{As}	1.523(1)	1.526(1)	1.528(1)	1.529(1)	1.528(1)	1.528(1)
Angle [°]	As-M(2)-As 1	106.03(6)	105.94(6)	105.91(6)	105.93(6)	105.98(6)	106.07(6)
	As-M(2)-As 2	111.219(31)	111.267(32)	111.279(31)	111.273(32)	111.244(33)	111.199(33)

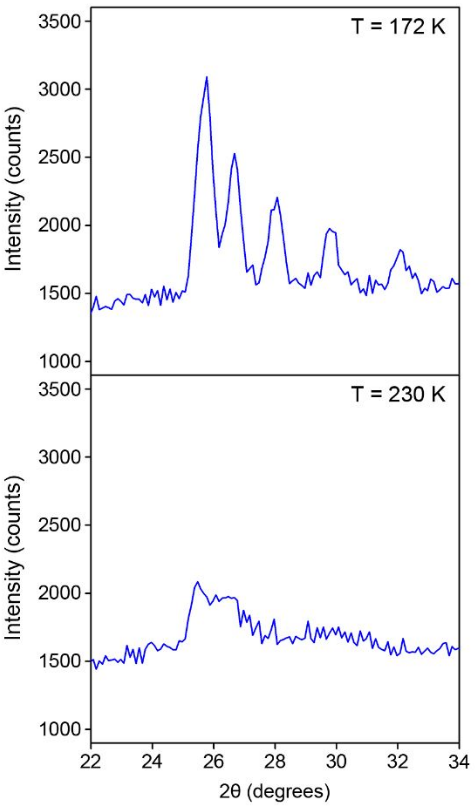

Figure S4: Selected high-intensity (D1B) diffraction patterns showing the magnetic reflections from the M(1) moments' magnetic structure. Above, at T = 172 K, long range order is observed, and both magnetic phases (with $k_2 = (½, ½, 0)$ and $k_3 = (½, ½, ½)$) are observed. Below, at T = 230 K, only short-range magnetic order is observed.

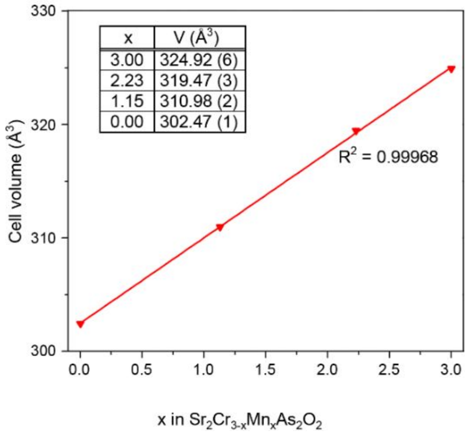

Figure S5: Variation of cell volumes with the Cr:Mn ratio in the $Sr_2Cr_{3-x}Mn_xAs_2O_2$ solid solution series. The table inset represents the values which were used in the graph - these were obtained from Jiang et al. for $Sr_2Cr_3As_2O_2$ (T = 298 K)[17], from this work for $Sr_2Cr_{1.15}Mn_{1.85}As_2O_2$ (T = 300 K), from Lawrence et al. for $Sr_2Cr_{0.77}Mn_{2.23}As_2O_2$ (T = 300 K)[18], and from Brock et al. for $Sr_2Mn_3As_2O_2$ (T = 300 K)[14]. The red line represents the simple linear regression fit of the data, with the coefficient of determination (R^2) value for this fit given on the right side.

Table S3: Crystallographic data table

**Source**	Neutron (constant wavelength)
**Chemical formula**	$Sr_2Cr_{1.85}Mn_{1.15}As_2O_2$
**Formula weight**	516.4535
**Temperature (K)**	300
**Crystal System**	Tetragonal
**Space group**	*I4/mmm* (no. 139)
***a* (Å)**	4.04379(5)
***c* (Å)**	18.9977(4)
**V (Å^3)**	310.654(7)
Z	2
***d*-space range (Å)**	0.86 – 11.91
χ^2	3.46
R_P	4.89
R_{WP}	3.88
**Definition of R factors**	$R_p = \Sigma\|y_i(obs) - y_i(calc)\|/\Sigma y_i(obs)$;<br>$R_{wp} = \{\Sigma w_i[y_i(obs) - y_i(calc)]^2/\Sigma w_i y_i(obs)^2\}^{1/2}$
